# Supplementary material for: Turkish adaptation of a new scale for measuring transactional distance between students and the learning technology
Source: PLoS One. 2025 Sep 24;20(9):e0331789. doi: 10.1371/journal.pone.0331789 (PMC12459776; doi:10.1371/journal.pone.0331789)
Supplement: S1 Appendix — (DOCX) [file pone.0331789.s001.docx]

**APPENDIX – 1: Technical Report of the new TDSTECH's Adaptation Study to Turkish Culture**.

| **Technical Report of the new TDSTECH Adaptation Process to Turkish Culture** | | | | | | | |  |
| --- | --- | --- | --- | --- | --- | --- | --- | --- |
| **Factor No** | **Item No** | **Item Total Correlations** | **Factor Loadings** | | **Factor Load./S.E.** | | **Cronbach Alpha** |  |
| **f1** | m1 | 0.521 – Sufficient | 0.645 – Sufficient | | 38.876 – Sufficient | | 0.674 – Acceptable |  |
|  | m2 | 0.495 – Sufficient | 0.546 – Sufficient | | 30.544 – Sufficient | |  |  |
|  | m3 | 0.666 – Sufficient | 0.792 – Sufficient | | 69.930 – Sufficient | |  |  |
| **f2** | m4 | 0.710 – Sufficient | 0.886 – Sufficient | | 124.029 – Sufficient | | 0.938 – Sufficient |  |
|  | m5 | 0.724 – Sufficient | 0.937 – Sufficient | | 172.622 – Sufficient | |  |  |
|  | m6 | 0.709 – Sufficient | 0.920 – Sufficient | | 154.998 – Sufficient | |  |  |
| **f3** | m7 | 0.722 – Sufficient | 0.875 – Sufficient | | 114.086 – Sufficient | | 0.837– Sufficient |  |
|  | m8 | 0.659 – Sufficient | 0.823 – Sufficient | | 82.587 – Sufficient | |  |  |
| **f4** | m9 | 0.830 – Sufficient | 0.939 – Sufficient | | 189.083 – Sufficient | | 0.857– Sufficient |  |
|  | m10 | 0.810 – Sufficient | 0.905 – Sufficient | | 138.446 – Sufficient | |  |  |
|  | m11 | 0.653 – Sufficient | 0.619 – Sufficient | | 37.953 – Sufficient | |  |  |
| **Fit Index** | **Result** | **Cut-Off** | **Interpretation** | | | | |  |
| RMSEA | 0.079  (0.073 - 0.085) | <0.08 | Intermediate | | | | |  |
| CFI | 0.964 | >0.95 | Good | | | | |  |
| TLI | 0.947 | >0.95 | Good | | | | |  |
| SRMR | 0.049 | <0.06 | Good | | | | |  |
| Chi-Square/df | 15.150 | <2 | Poor | | | | |  |
| **Heterotrait-monotrait ratio of correlations – 2 (HTMT2)** | | | | | | | |  |
|  | **f1** | **f2** | | **f3** | | **f4** | |  |
| **f1** | 1.000 | 0.601  *(<0.90-Sufficient)* | | 0.746  *(<0.90-Sufficient*) | | 0.879  *(<0.90-Sufficient)* | |  |
| **f2** |  | 1.000 | | 0.556  *(<0.90-Sufficient)* | | 0.743  *(<0.90-Sufficient)* | |  |
| **f3** |  |  | | 1.000 | | 0.884  *(<0.90-Sufficient)* | |  |
| **f4** |  |  | |  | | 1.000 | |  |
| f1: Learner Readiness For Using Technology; f2: Effectiveness; f3: Efficiency; f4: Satisfaction | | | | | | | |  |
|  |  |  |  |  |  |  |  |  |
