# Supplementary material for: Turkish adaptation of a new scale for measuring transactional distance between students and the learning technology
Source: PLoS One. 2025 Sep 24;20(9):e0331789. doi: 10.1371/journal.pone.0331789 (PMC12459776; doi:10.1371/journal.pone.0331789)
Supplement: S2 Appendix — (DOCX) [file pone.0331789.s002.docx]

**APPENDIX – 2: Application Guide for the new TDSTECH adapted.**

**Number of items:** 11

**Number of dimensions:** 4

**The scoring system**: 5-point scoring system

**Reverse items:** y7 and y8

**Estimated application time:** 3 – 7 minutes

**Form of application:** Online or paper-pencil

**Forced choice items in the online form:** Unadvisable

**Distribution of the items to the dimensions:**

| Item code | Four-Dimension Construct |
| --- | --- |
| y1 | f1: Learner Readiness For Using Technology |
| y2 |  |
| y3 |  |
| y4 | f2: Effectiveness |
| y5 |  |
| y6 |  |
| y7 | f3: Efficiency |
| y8 |  |
| y9 | f4: Satisfaction |
| y10 |  |
| y11 |  |

**The instructional text:**

Dear Participant,

In this scale that you are participating in the application, you will be referred to items that relate to transactional distance. Transactional distance is defined as "the gap between a teacher's (or teaching team's) understanding and that of a learner" (M. G. Moore, 2018). In addition, some personal information* (gender, grade level, department, etc.) is asked, which is used for comparisons and equivalence analyses. the 11 items in the scale are rated on a 5-point Likert scale. After reading the items, it is enough to mark the category that comes closest to your opinion. This application takes a total of 3-7 minutes. There will be no academic grading after this application. In addition, your responses will not be shared with other individuals and/or institutions without your consent. Your answers will be securely stored by the researchers. You may request that your responses be removed from the analysis at any time by contacting the researchers. Thank you for your participation.

**May differ depending on the purpose of the research.*

Please confirm that you have read and understood the above text by ticking the box below.

I have read and understand

**Contact Information of researchers:**

Name – Surname:

E-mail :

Phone number :

Adress :
