# Supplementary material for: Turkish adaptation of a new scale for measuring transactional distance between students and the learning technology
Source: PLoS One. 2025 Sep 24;20(9):e0331789. doi: 10.1371/journal.pone.0331789 (PMC12459776; doi:10.1371/journal.pone.0331789)
Supplement: Attachment — Submitted filename: PONE-D-25-35376_reviewer....pdf [file pone.0331789.s003.pdf]

PLOS ONE

Turkish Adaptation of The New TDSTECH Scale

--Manuscript Draft--

**Manuscript Number:**	PONE-D-25-35376
**Article Type:**	Research Article
**Full Title:**	Turkish Adaptation of The New TDSTECH Scale
**Short Title:**	Adaptation of The New TDSTECH
**Corresponding Author:**	Alperen YANDI<br>BAİBÜ: Bolu Abant Izzet Baysal Universitesi<br>Bolu Merkez, Bolu TÜRKIYE
**Keywords:**	Transactional distance, distance learning, the new scale for measuring transactional distance, scale adaptation
**Abstract:**	It is important to examine the concept of transactional distance in order to identify potential areas for improvement in the distance education process. A variety of measurement tools are available, each with distinct features for assessing this structure. Among these scales, the scale that offers the opportunity to examine transactional distance based on the most up-to-date theoretical framework is the new TDSTECH. This scale was developed by Weidlich and Bastiaens (2018). The universality of distance education principles, regardless of cultural differences, allows for a more comprehensive examination of the structures that emerge in this field. In order to achieve this, it is essential that existing measurement tools are adapted to cultures with different features. Adaptation studies ensure that the features to be measured are addressed in the context of different cultural structures and that reflections from students with different learning experiences can be considered. In this context, this study focuses on adapting "the new scale for measuring transactional distance between students and the learning technology" to Turkish culture. During the adaptation process, the 18-step procedure recommended by the International Test Commission was meticulously followed. The scale was administered to two study groups. Linguistic equivalence, pre-validity, and reliability examinations were performed using the data from study group 1. Data from study group 2 were utilized for further reliability, construct, and discriminant validity analyses. The results of these analyses indicated that the scale is suitable for use in Turkish culture. This study is expected to contribute significantly to enhancing the efficiency of distance learning programs within Turkish culture by providing a validated tool for assessing transactional distance.
**Order of Authors:**	Alperen YANDI
**Additional Information:**	
**Question**	**Response**
**Financial Disclosure**<br><br>Enter a financial disclosure statement that describes the sources of funding for the work included in this submission. Review the submission guidelines for detailed requirements. View published research articles from *PLOS ONE* for specific examples.<br><br>This statement is required for submission and **will appear in the published article** if the submission is accepted. Please make	The author(s) received no specific funding for this work.

Powered by Editorial Manager® and ProduXion Manager® from Aries Systems Corporation

sure it is accurate.<br><br>**Funded studies**<br>Enter a statement with the following details:<br>• Initials of the authors who received each award<br>• Grant numbers awarded to each author<br>• The full name of each funder<br>• URL of each funder website<br>• Did the sponsors or funders play any role in the study design, data collection and analysis, decision to publish, or preparation of the manuscript?<br><br>Did you receive funding for this work?	
**Competing Interests**<br><br>Use the instructions below to enter a competing interest statement for this submission. On behalf of all authors, disclose any competing interests that could be perceived to bias this work—acknowledging all financial support and any other relevant financial or non-financial competing interests.<br><br>This statement is required for submission and **will appear in the published article** if the submission is accepted. Please make sure it is accurate and that any funding sources listed in your Funding Information later in the submission form are also declared in your Financial Disclosure statement.<br><br>View published research articles from *PLOS ONE* for specific examples.	The authors have declared that no competing interests exist.

**NO authors have competing interests**<br><br>Enter: *The authors have declared that no competing interests exist.*<br><br>**Authors with competing interests**<br><br>Enter competing interest details beginning with this statement:<br><br>*I have read the journal's policy and the authors of this manuscript have the following competing interests: [insert competing interests here]*<br><br>* typeset	
**Ethics Statement**<br><br>Enter an ethics statement for this submission. This statement is required if the study involved:<br><br>• Human participants<br>• Human specimens or tissue<br>• Vertebrate animals or cephalopods<br>• Vertebrate embryos or tissues<br>• Field research<br><br>Write "N/A" if the submission does not require an ethics statement.<br><br>General guidance is provided below. Consult the submission guidelines for detailed instructions. **Make sure that all information entered here is included in the Methods section of the manuscript.**	This study received approval from the Ethics Committee of Bolu Abant Izzet Baysal University (Approval Number: NO. 2021/430 [29.11.2021 and the meeting number: 2021/11]), and all procedures adhered to the ethical standards set by institutional and national research committees, along with the 1964 Helsinki Declaration and its subsequent amendments. Before participation, all participants received a written online information sheet outlining the study's goal, the voluntary nature of participation, data confidentiality, and the possibility to withdraw at any time without penalty. Participants electronically supplied written informed permission by actively signaling their acceptance using the online survey system prior to completing the questionnaire. Only the data from participants who granted this consent were incorporated into the study. This research did not include minors; hence, parental or guardian consent was unnecessary.

Powered by Editorial Manager® and ProduXion Manager® from Aries Systems Corporation

**Format for specific study types**<br><br>**Human Subject Research (involving human participants and/or tissue)**<br>• Give the name of the institutional review board or ethics committee that approved the study<br>• Include the approval number and/or a statement indicating approval of this research<br>• Indicate the form of consent obtained (written/oral) or the reason that consent was not obtained (e.g. the data were analyzed anonymously)<br><br>**Animal Research (involving vertebrate animals, embryos or tissues)**<br>• Provide the name of the Institutional Animal Care and Use Committee (IACUC) or other relevant ethics board that reviewed the study protocol, and indicate whether they approved this research or granted a formal waiver of ethical approval<br>• Include an approval number if one was obtained<br>• If the study involved *non-human primates*, add *additional details* about animal welfare and steps taken to ameliorate suffering<br>• If anesthesia, euthanasia, or any kind of animal sacrifice is part of the study, include briefly which substances and/or methods were applied<br><br>**Field Research**<br><br>Include the following details if this study involves the collection of plant, animal, or other materials from a natural setting:<br>• Field permit number<br>• Name of the institution or relevant body that granted permission	
**Data Availability**<br><br>Authors are required to make all data underlying the findings described fully available, without restriction, and from the time of publication. PLOS allows rare exceptions to address legal and ethical concerns. See the PLOS Data Policy and FAQ for detailed information.	Yes - all data are fully available without restriction

Powered by Editorial Manager® and ProduXion Manager® from Aries Systems Corporation

A Data Availability Statement describing where the data can be found is required at submission. Your answers to this question constitute the Data Availability Statement and **will be published in the article**, if accepted.<br><br>**Important:** Stating 'data available on request from the author' is not sufficient. If your data are only available upon request, select 'No' for the first question and explain your exceptional situation in the text box.<br><br>Do the authors confirm that all data underlying the findings described in their manuscript are fully available without restriction?	
**Describe where the data may be found in full sentences. If you are copying our sample text, replace any instances of XXX with the appropriate details.**<br><br>• If the data are **held or will be held in a public repository**, include URLs, accession numbers or DOIs. If this information will only be available after acceptance, indicate this by ticking the box below. For example: *All XXX files are available from the XXX database (accession number(s) XXX, XXX.).*<br>• If the data are all contained **within the manuscript and/or Supporting Information files**, enter the following: *All relevant data are within the manuscript and its Supporting Information files.*<br>• If neither of these applies but you are able to provide **details of access elsewhere**, with or without limitations, please do so. For example:<br><br>*Data cannot be shared publicly because of [XXX]. Data are available from the XXX Institutional Data Access / Ethics Committee (contact via XXX) for researchers who meet the criteria for access to confidential data.*<br><br>*The data underlying the results presented in the study are available from (include the name of the third party*	THE DATASETS URL:<br><br>https://drive.google.com/drive/folders/18OQC_-qcSWYHAMPm4ec-nKy8nJel_nLS?usp=sharing

Powered by Editorial Manager® and ProduXion Manager® from Aries Systems Corporation

and contact information or URL).<br>• This text is appropriate if the data are owned by a third party and authors do not have permission to share the data.<br><br>* typeset	
Additional data availability information:	Tick here if the URLs/accession numbers/DOIs will be available only after acceptance of the manuscript for publication so that we can ensure their inclusion before publication.

Powered by Editorial Manager® and ProduXion Manager® from Aries Systems Corporation

Manuscript Click here to access/download;Manuscript;Manuscript3.docx

TITLE PAGE

The researcher, who is an assistant professor in the Department of Educational Measurement and Evaluation at his institution, continues his studies of measurement equivalence, differential item functioning, and the examination of various psychological constructs.

Correspondence Author: Alperen YANDI

Faculty of Education, Bolu Abant Izzet Baysal University: Golkoy Campus 14030 – Bolu/Turkey

alperenyandi@ibu.edu.tr

+905056758867

+903742541000-1763

Turkish Adaptation of The New TDSTECH Scale

ABSTRACT

It is important to examine the concept of transactional distance in order to identify potential areas for improvement in the distance education process. A variety of measurement tools are available, each with distinct features for assessing this structure. Among these scales, the scale that offers the opportunity to examine transactional distance based on the most up-to-date theoretical framework is the new TDSTECH. This scale was developed by Weidlich and Bastiaens (2018). The universality of distance education principles, regardless of cultural differences, allows for a more comprehensive examination of the structures that emerge in this field. In order to achieve this, it is essential that existing measurement tools are adapted to cultures with different features. Adaptation studies ensure that the features to be measured are addressed in the context of different cultural structures and that reflections from students with different learning experiences can be considered. In this context, this study focuses on adapting “the new scale for measuring transactional distance between students and the learning technology” to Turkish culture. During the adaptation process, the 18-step procedure recommended by the International Test Commission was meticulously followed. The scale was administered to two study groups. Linguistic equivalence, pre-validity, and reliability examinations were performed using the data from study group 1. Data from study group 2 were utilized for further reliability, construct, and discriminant validity analyses. The results of these analyses indicated that the scale is suitable for use in Turkish culture. This study is expected to contribute significantly to enhancing the efficiency of distance learning programs within Turkish culture by providing a validated tool for assessing transactional distance.

Keywords: Transactional distance, distance learning, the new scale for measuring transactional distance, scale adaptation

Biography

The researcher is working as an assistant professor at the Division of Educational Measurement and Evaluation, Bolu Abant Izzet Baysal University in Turkey.

INTRODUCTION

The field of distance learning has a long historical precedent, with documented instances dating back to the 20th century (Sumner, 2000). One significant factor contributing to this increase is the adoption of distant or hybrid instructional methods during the pandemic caused by the SARS-CoV-2 virus. It is, however, important to distinguish between fully planned and designed distance learning experiences and the courses offered online in response to the crisis, which have been referred to as "emergency remote teaching" (S. Moore & Hodges, 2023). It should be noted that the principles of instructional design specific to distance education were not necessarily applied in the context of emergency remote teaching. The shift to online education, accelerated by the COVID-19 pandemic, has significantly influenced the growth of distance learning. The rapid transition from traditional classrooms to online learning environments was necessitated by the pandemic, prompting extensive research and adaptation in educational practices (Sato et al., 2024). The data from the National Center for Education Statistics and the National Council for State Authorization Reciprocity Agreements illustrate a substantial increase in online enrollments, highlighting the trend toward distance education. Additionally, systematic reviews indicate that the integration of educational technology and innovative teaching strategies played a crucial role in this shift, underscoring the necessity for continuous examination of the quality and efficacy of distance learning (Koh & Daniel, 2022). A number of studies have examined the factors affecting the quality of distance learning and the reasons for student attrition in such programmes (Croxton, 2014; Joo et al., 2011; Kauffman, 2015; Lee & Choi, 2013). Among the most critical factors is technology, as students' proficiency in interacting with technology can significantly influence the effectiveness of learning by affecting other forms of interaction (student-student, student-teacher) during the instructional process (Hillman et al., 1994).

Students' interactions with technology can be related to "transactional distance theory." Transactional distance theory provides a broad framework for describing variables in distance education. For this reason, it is explained that transactional distance is a construct that governs distance education and influences teaching and learning processes (M. G. Moore, 2018). According to this theory, based on "structure", "dialog," and "autonomy," transactional distance is defined as "the gap between a teacher's (or teaching team's) understanding and that of a learner." In the case of distance education, it is the process of dialog between teachers and learners and structuring the curriculum to minimize this gap through technology (M. G. Moore, 2018).

The concept of structure, which is one of the components of operational distance, pertains to the flexibility level of all elements that constitute a course, including the programme, teaching strategy and evaluation. A further component, the concept of dialogue, is defined as a communication environment created in an open climate. In this environment, two or more participants share their questions and answers, opinions and directions. Depending on the use of different technologies, the dialog opportunities in the instructional process also vary (Burbules, 1993). The final component of transactional distance, student autonomy, is associated with the degree of student involvement in the learning process. This component provides information regarding the extent to which students are able to participate in the design, implementation, and evaluation of the programme. The research conducted by M. G. Moore (1973, 1976, cited by M. G. Moore, 2018) has demonstrated that the three components of transactional distance, namely structure, dialogue and student autonomy, interact with one another. It has been asserted that this interaction between these three concepts indicates the flexibility of the teaching process and the pedagogical complexity of distance education (Peters, 2013).

Conceptualized as a psychometric construct based on the analysis of the interaction between structure, dialogue, and autonomy, transactional distance has been measured via self-report in several studies (Ekwunife-Orakwue & Teng, 2014; Goel et al., 2012; Paul et al., 2015; Weidlich & Bastiaens, 2018). The negative effects of high transactional distance on learning (Zhang, 2003) necessitated a more sensitive examination of this construct. Accordingly, transactional distance is divided into sub-dimensions such as student-student, student-teacher, and student-content (M. G. Moore, 2018). For these dimensions, the higher the interaction between the components, the lower the transactional distance. In this way, meaningful learning is supported (Anderson, 2003). Hillman et al. (1994) noted that the relationship between technology and distance education is neglected even though it is an important component of the instructional process. With the emergence of this situation, student-interface interaction has begun to be considered as another sub-dimension to show the effect of technology in determining transactional distance. This sub-dimension focuses on users' interpretations of the perceived and visible aspects of the interface. Users attempt to understand the interface by predicting its future behaviors and controlling their actions accordingly. Y. J. Chen (2001) and Zhang (2003) developed measurement tools to evaluate the transactional distance between students and technology interfaces (TDSTECH). However, the relevance of these and other measurement tools has become debatable due to rapid

technological advancements. Indeed, Paul et al. (2015) noted that many participants tended to provide positive responses when using these tools.

The need to update the content of the items in these measurement instruments led researchers to develop new measurement instruments. For example, Weidlich and Bastiaens (2018) developed a measurement instrument that captures the perceived transaction distance between the student and the technology under the dimensions of " the student's basic knowledge of using the required technology" (1) and " the design and functionality (e.g., ease of use)" (2). In developing the scale, the "Online Learning Readiness Scale" developed by Hung et al. (2010) was used for the first dimension and the concept of "usability" defined by the International Organization for Standardization (ISO) was used for the second dimension. In forming the first dimension, the items of the "Computer and Internet Self-Efficacy" dimension were arranged in the Online Learning Readiness Scale. This dimension is about assessing an individual's perception and ability in using technology. The second dimension is to measure "the extent to which a product can be used by specific users to achieve specific goals with effectiveness, efficiency, and satisfaction in a specific context of use - usability" (Ergonomic requirements for office work with visual display terminals [VDTs], n.d.).

In order to enhance the quality of distance education across diverse cultural contexts, it is essential to conduct adaptation studies of the latest scales that encompass the student-interface interaction dimension. Upon examination of studies on Turkish culture, it becomes evident that the 'Transactional Distance Scale', initially developed by Zhang (2003), has been adapted (Yılmaz & Keser, 2015). Furthermore, Horzum (2011) developed a scale comprising 38 items and five sub-factors for measuring transactional distance. The scale, which is based on the transactional distance theory, comprises five sub-factors, as follows: These include dialogue, autonomy, structure flexibility, content organisation and student control. The use of outdated transactional distance measurements in Turkish culture gives rise to questions about the appropriateness of the results. Furthermore, the rapid advancement of technology and the impact of the pandemic on distance learning have highlighted the necessity to re-evaluate the transactional distance from a contemporary standpoint. In this way, it can contribute to the more precise organization of the distance learning process.

The aim of this study is to adapt "The New TDSTECH" instrument, which was developed by Weidlich and Bastiaens (2018) and includes a dimension that has been shown to need to be examined up-to-date, such as student-interface interaction, to Turkish culture. Since the validity and reliability evidence of the scale have been provided, it was decided to conduct an

adaptation study instead of developing a new one. The reasons supporting this choice are as follows: Firstly, the scale includes the critical dimension of student-interface interaction, which is fundamental to assessing transactional distance in contemporary contexts. Secondly, despite the existence of discrepancies between the second cultural structures, technological applications frequently exhibit shared characteristics, thereby enabling the utilisation of instruments developed in disparate cultural structures following adaptation. Thirdly, the development of a new scale may be disadvantageous in terms of usefulness, as it requires extensive time and resources for validation and reliability testing, which could result in delays to its implementation and practical application (Hambleton & Patsula, 1999). To illustrate, the process of developing, testing and refining a new scale can take years, during which time the rapid pace of technological advancement may render the new scale obsolete by the time it is ready for use.

The linguistic and structural equivalence of measurement instruments in different cultures provides the foundation for accurate and valid cross-cultural research. Consequently, international research collaborations will be facilitated, allowing for a more comprehensive examination of the structures in question. These advantages underscore the practicality, efficiency, and wider applicability of adapting an existing scale instead of developing a new scale. In light of these considerations, the research question is formulated as follows:

What is the reliability and validity of the four-factor structure of the new TDSTECH in Turkish culture?

METHOD

This section contains information about the steps of adaptation process. In this way, it aims to provide other researchers with a detailed of the process. It also includes information regarding the characteristics of the sample and the adapted measurement instrument. Furthermore, it encompasses all analysis procedures conducted during the adaptation steps.

The Adaptation Process

This research was conducted using a mixed methods approach. In order to guarantee the reliability, validity and cultural equivalence of the scale, qualitative and quantitative methods were integrated during the research process, in accordance with the established scale adaptation steps.

In the process of adaptation, the 18-step procedure recommended by the International Test Commission (ITC) was considered. 3 of the steps followed are pre-processing (PC), 5 are test development (TD) and the adaptation process, and 4 are obtaining empirical findings (C). Process administration (A), scoring and interpretation (SSI), and documentation (Doc) are comprised of two distinct steps. (ITC Guidelines for Translating and Adapting Tests, 2018). The individual steps are as follows:

> *"PC-1 (1) Obtain the necessary permission from the holder of the intellectual property rights relating to the test before carrying out any adaptation.*
>
> *PC-2 (2) Evaluate that the amount of overlap in the definition and content of the construct measured by the test and the item content in the populations of interest is sufficient for the intended use (or uses) of the scores.*
>
> *PC-3 (3) Minimize the influence of any cultural and linguistic differences that are irrelevant to the intended uses of the test in the populations of interest.*
>
> *TD-1 (4) Ensure that the translation and adaptation processes consider linguistic, psychological, and cultural differences in the intended populations through the choice of experts with relevant expertise.*
>
> *TD-2 (5) Use appropriate translation designs and procedures to maximize the suitability of the test adaptation in the intended populations.*
>
> *TD-3 (6) Provide evidence that the test instructions and item content have similar meaning for all intended populations.*

TD-4 (7) Provide evidence that the item formats, rating scales, scoring categories, test conventions, modes of administration, and other procedures are suitable for all intended populations.

TD-4 (7) Provide evidence that the item formats, rating scales, scoring categories, test conventions, modes of administration, and other procedures are suitable for all intended populations.

TD-5 (8) Collect pilot data on the adapted test to enable item analysis, reliability assessment and small-scale validity studies so that any necessary revisions to the adapted test can be made.

C-1 (9) Select sample with characteristics that are relevant for the intended use of the test and of sufficient size and relevance for the empirical analyses.

C-2 (10) Provide relevant statistical evidence about the construct equivalence, method equivalence, and item equivalence for all intended populations.

C-3 (11) Provide evidence supporting the norms, reliability, and validity of the adapted version of the test in the intended populations.

C-4 (12) Use an appropriate equating design and data analysis procedures when linking score scales from different language versions of a test.

A-1 (13) Prepare administration materials and instructions to minimize any culture- and language-related problems that are caused by administration procedures and response modes that can affect the validity of the inferences drawn from the scores.

A-2 (14) Specify testing conditions that should be followed closely in all populations of interest

SSI-1 (15) Interpret any group score differences with reference to all relevant available information.

SSI-2 (16) Only compare scores across populations when the level of invariance has been established on the scale on which scores are reported.

Doc-1 (17) Provide technical documentation of any changes, including an account of the evidence obtained to support equivalence, when a test is adapted for use in another population.

Doc-2 (18) Provide documentation for test users that will support good practice in the use of an adapted test with people in the context of the new population."

An attempt was made to include all 18 steps except the 12th and 16th steps. For the 12th and 16th steps, the data set of the scale from groups with different cultures and languages is needed. However, these steps were not possible because it was not possible to obtain such data for a large-scale application and the data used in the development of the original scale had limited release by the researchers. With the large-scale applications that can be realized, follow-up studies can be conducted for these two steps. The findings obtained in the other steps are detailed in the Results section.

Study Groups

For the adaptation process, data were collected from two different study groups. The first was Study Group 1, in which the pilot application was conducted to ensure linguistic equivalence and identify possible arrangements; the other is Study Group 2, which was formed to obtain empirical evidence for the adapted scale.

Study Group 1

A smaller number of participants was specified for this study group than for the other study group. 46 4th grade students from the foreign language teaching department of a university in Turkey (study period 4 years) were included in this study group. The age and gender distribution of the participants in this group is shown in Table 1.

Table 1. Demographic information of study group 1

**Gender**	**Frequencies**	**Percentage**
**Female**	36	78.30
**Male**	10	21.70
**Total**	46	100.00
**Age**	**Frequencies**	**Percentage**
**21**	3	6.52
**22**	27	58.70
**23**	10	21.74
**24**	2	4.35
**25**	2	4.35
**31**	2	4.35
**Total**	46	100.00
**Mean**	22.760	
**Standard Deviation**	1.968	

From the results shown in Table 1, the age range of the participants in the study group is between 21 and 31 years. Another piece of information about the age is that many of the participants are 22 years old (58.70%). The mean age of the participants was calculated to be 22.760 ± 1.968. In addition, the number of female participants was higher in study group 1 (78.30%). The reason why the participants in study group 1 were selected from the foreign language education department is that they have high proficiency in the source and target languages.

The study group 2

Study group 2 consists of 2402 students studying at a university in Turkey. The distribution of these students pursuing their education in different faculties for the variables of course of study, age, and gender is given in Table 2.

Table 2. Demographic information of study group 2

**Gender**	**Frequencies**	**Percentage**
**Female**	1608	66.90
**Male**	794	33.10
**Total**	2402	100.00
**Personal Computer**	**Frequencies**	**Percentage**
**Having**	1983	82.60
**Not Having**	419	17.40
**Total**	2402	100.00
**Study Period**	**Frequencies**	**Percentage**
**Associate degree**	300	12.50
**Scientiæ Baccalaureus**	2102	87.50
**Total**	2402	100.00
**Age**	**Frequencies**	**Percentage**
17	2	0.10
18	67	2.80
19	198	8.20
20	466	19.40
21	507	21.10
22	461	19.20
23	240	10.00
24	129	5.40
25	74	3.10
26	33	1.40
27	28	1.20
28	29	1.20
29	19	0.80
30	9	0.40
31	13	0.50
32	11	0.50

33	7	0.30
34	13	0.50
35	14	0.60
36	11	0.50
37	13	0.50
38	9	0.40
39	11	0.50
40	16	0.70
41	9	0.40
42	6	0.20
45	7	0.30
**Total**	2402	100.00
**Mean**	22.470	
**Standard Deviation**	4.197	

The results in Table 2. show that the age range of the participants is between 17 and 45 years. The average age of the participants was calculated to be 22.470 ± 4.197. It also shows that the number of females (66.90%) in the study group is higher than the number of males (33.10%). It was found that 87.50% of the participants were studying in Scientiæ Baccalaureus programs and 12.50% were studying in Associate degree programs. Participants in study group 2 were also asked about the item on owning a personal computer. Based on the results of this item, which provides a more detailed explanation of the characteristics of the study group, it was determined that 82.60% of the participants own a personal computer.

Measurement Instrument

The new TDSTECH consists of 11 items and was developed by Weidlich and Bastiaens (2018). The items of the new TDSTECH were prepared by compiling the TDSTECH items developed by Hillman et al. (1994). The new TDSTECH is based on the assumption that transactional distance is created by the interaction between the dimensions of "the student's basic mastery of the necessary technology" and "the design and functionality (e.g., ease of use) of the technology itself as perceived by the student." In preparing the item pool for the scale, items from the Computer & Internet self-efficacy scale developed by Hung et al. (2010) were used for the first dimension. The phrase "software for online learning" in the items was replaced with "Moodle". For the second dimension, items were created for the concept of ease of use defined by the "International Organization for Standardization". As a result of the principal component analysis conducted on the item pool, it was found that the items were grouped under four components. These four components are referred to as "learner readiness to use technology," "effectiveness," "efficiency," and "satisfaction." After one of the items

was removed from the item pool because it was factor loaded under another component, 11 items remained in the final form. The scale, in which two items (7-8) in the "efficiency" dimension are negative, has a 5-point scoring system. The highest scoring category is "strongly agree"; the lowest scoring category was defined as "strongly disagree." The factor loadings of the items range from 0.588 - 0.939. In the final form, the dimensions of learner readiness to use the technology, effectiveness, and satisfaction consist of 3 items, and the efficiency dimension consists of 2 items. The Cronbach Alpha coefficient calculated by the developers for the scale is also high (α = 0.873) (Weidlich & Bastiaens, 2018). The item distributions of the four-component construct can be found in Table 3.

Table 3. Distribution of the items for the four-component construct of the new TDSTECH.

Item code	Four-Component Construct
y1	Component 1 (C1)
y2	
y3	
y4	Component 2 (C2)
y5	
y6	
y7	Component 3 (C3)
y8	
y9	Component 4 (C4)
y10	
y11	

The scale, whose dimensional construct is shown in Table 3, was transferred to the online form after translation to reach more participants in less time. The application took 4-6 minutes, depending on the reading and marking speed of the participants.

Analysis of the data

Several analyses of the data were conducted in the adaptation study. Prior to all analyses, items 7 - 8, which were negative, were recoded. In addition, the significance level for all analyses was 0.05. Item statistics, correlation analyses, and hypothesis tests were performed for the data in study group 1. In determining the technique to use for correlation analysis and hypothesis testing, the normal distribution of the study group 1 results was tested using the Shapiro-Wilks test. According to these test results, the calculation of Pearson's correlation coefficient and the T-test for independent samples were performed. The purpose of these two analyses was to check for linguistic equivalence. In addition, the item-total correlations for the

items were evaluated. A threshold of 0.300 was assumed for the item-total correlations (Field, 2009). The results of these analyses are presented under the headings of steps 6 - 8 and 15 in the Results section.

Confirmatory factor analysis (CFA) was conducted on the data from Study Group 2 to establish the structural validity of the scale. Prior to CFA, potential outliers in the data set were checked. When checking for outliers, each item score was converted to a Z-standard score; participants who scored outside the range of ± 3.290 on any item were excluded from the data set as outliers (Field, 2009). In this analysis, 95 participants were identified as outliers. The analysis process continued with data from the remaining 2307 individuals. Subsequently, multivariate outlier detection was conducted using the Mahalanobis distance (De Maesschalck et al., 2000). The significance of the calculated Mahalonobis distances was checked through a chi-square test. The value of 0.001 was taken as the critical value for this test (Mardia, 1974). Following this test, 38 participants identified as extreme values were excluded from the data set, resulting in a final sample size of 2269 individuals prior to analysis.

Following the arrangements of the data set, the univariate and multivariate normality, linearity and multicollinearity/singularity assumptions were investigated. For univariate normality, the standardised values of the skewness and kurtosis coefficients were examined for each item. In order to interpret these standardised values, a threshold value of 1.96 suggested for large samples was employed. The selection of 1.96 as the cut-off value is consistent with a 95% confidence interval. Accordingly, standardised values within the range of ±1.96 are deemed to be normal at a 5% statistical significance level (Altman & Bland, 1995; Field, 2009). In the examinations, the standard values for the skewness of all items were observed to range between -39.963 and 7.660. The standard kurtosis values ranged between -7.640 and 21.604. Upon examination of the standard values for skewness and kurtosis of the items, it was determined that a subset of items exhibited a non-normal distribution. Furthermore, Mardia's tests (1974) were employed to assess multivariate normality. The results of these tests indicated that multivariate normality could not be achieved for the data set. This finding was taken into account when selecting the estimation method and matrix type to be employed in the analysis processes. Accordingly, the correlation matrix and robust maximum likelihood (MLR) estimation method were selected as the preferred approach. In cases where the normality assumption is violated, MLR is one of the recommended estimation methods (Gana & Broc, 2018).

In order to examine the linearity of the data, scatter plots were utilised. Furthermore, Bartlett's Sphericity test was employed .($\chi2(55) = 18275.057$; $p < 0.05$) (Tabachnick et al., 2013). The results demonstrate that the relationship between the items is linear. Finally, the Variance Inflation Factor (VIF), and tolerance values were calculated in order to check the assumption of multicollinearity. In order to ascertain that there is no multicollinearity, the VIF values must be less than 10 and the tolerance values must be greater than 0.10 (Kline, 2018; Tabachnick et al., 2013). The VIF values ranged between 1.380 and 5.200, while the tolerance values ranged between 0.1902 and 0.720. These results demonstrate that no multicollinearity issues exist.

Prior to utilising the Robust Maximum Likelihood (MLR) estimation method, it is essential to ascertain the veracity of the assumptions pertaining to the residuals. The initial assumption to be checked is the normality of the residual values. In order to examine this assumption, a Q-Q plot can be utilised (Kline, 2018). The second assumption is that the relation between residual values and predictive values are linearity. This assumption can be evaluated using scatter plots (Bollen, 1989). An additional crucial assumption is that the residual values have homoscedasticity for different level predicted values. The Breusch-Pagan (BP) test can be employed to assess this assumption (Halunga et al., 2017). The final assumption regarding the residuals that requires examination is that of autocorrelation. The Durbin-Watson test can be utilised to evaluate the presence of autocorrelation (Ali, 1987; Durbin & Watson, 1950). In the event that the results of the two aforementioned tests are not statistically significant, it can be inferred that the assumptions are validated.

The Q-Q plot, generated to assess the normality of the residuals, as illustrated in Figure 1, indicates that the central portion is approximately normally distributed. However, deviations in the tails, especially in the upper tail, indicate the existence of possible problems such as extreme values (Ghasemi & Zahediasl, 2012). Although the Q-Q plot shows that all the residuals are not normally distributed and this may affect the analysis results (Kline, 2018), the MLR estimation method is statistically robust against violations of the normality assumption (Li, 2016; Satorra & Bentler, 1994).

Figure 1. Q-Q plot of standardized residuals of CFA model

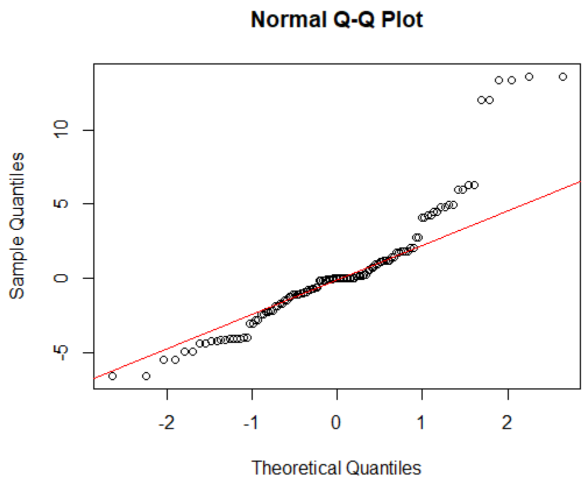

Figure 2 illustrates a scatter plot employed to assess the linearity of the residuals. To fulfill this assumption, the residuals must be randomly distributed around zero, exhibiting no discernible pattern (Osborne & Waters, 2002). In this plot, the residuals appear to be uniformly distributed across the range of predicted values. No discernible systematic pattern is in the plot, indicating that the linearity assumption is reasonably met (Kline, 2018).

Figure 2. Scatter plot of standardized residuals of CFA model

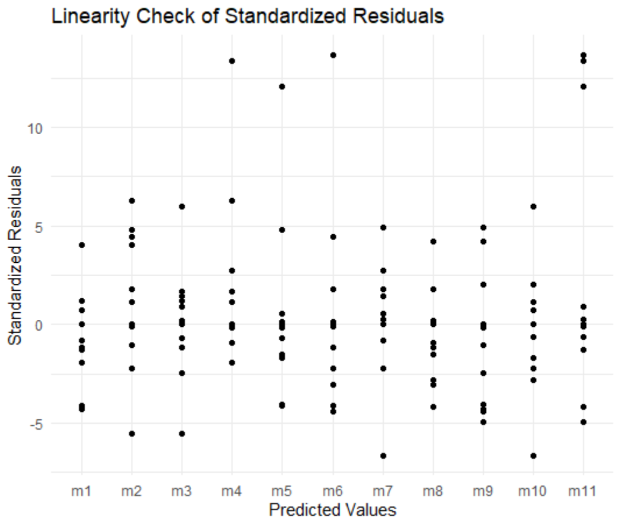

The Breusch-Pagan test statistic for homoscedasticity of residuals (BP = 3.6517, df = 1, p-value = 0.05601) indicated that the null hypothesis of no heteroscedasticity could not be rejected at the 0.05 level (Breusch & Pagan, 1979). The Durbin-Watson test statistic (DW = 1.7604, p-value = 0.09192) was employed to assess the presence of autocorrelation in the residuals of the confirmatory factor analysis model. The resulting value of 1.7604 is close to

the critical value of 2, indicating the absence of autocorrelation (Durbin & Watson, 1950). Although the p-value of 0.09192 is not below the traditional threshold of 0.05, it indicates marginal evidence against the null hypothesis of no positive autocorrelation. Therefore, the results indicate that there is no strong evidence of significant autocorrelation in the residuals (Field, 2009).

After testing the assumptions, the fit indices chi-square/sd, RMSEA, CFI, TLI, and SRMR were examined for structural validity. < 2 for chi-square/sd, < 0.08 for RMSEA, ≥ 0.95 for CFI and TLI, and < 0.06 for SRMR were considered criteria for good fit when interpreting the fit indices (Cole, 1987; Cudeck & Browne, 1983; Hu & Bentler, 1999). Discriminant validity analyses were also conducted for the scale. For this validity, the heterotrait-monotrait ratio of correlations-2 (HTMT2) was calculated (Roemer et al., 2021). A value of 0.90 was adopted as the threshold for the HTMT2 values (Gold et al., 2001; Teo et al., 2008). To determine the reliability of the scale, Cronbach's Alpha was evaluated for data from both study groups. The range of 0.60-0.70 was used as a criterion for reliability (George & Mallery, 2003). The results of all these analyses are presented under the heading of Step 11. SPSS 20.0, Mplus 7.3 package programs, and R 4.2.1. (R Core Team, 2022) were used for the analysis process. The calculateHTMT function in the cSEM package version 0.4.0 (Rademaker & Schuberth, 2020) was used in the analysis performed via R.

Furthermore, multi-group confirmatory factor analysis (MGCFA) was conducted to ascertain measurement invariance. In these analyses, the maximum likelihood estimation method was again selected. The results of the MGCFA are presented in detail in Step 10. In the MGCFA process, measurement invariance analyses were conducted in accordance with the stepwise testing method proposed by Meredith (1993). In this process, analysis were performed for the configural, metric and scalar invariance stages. Evidence regarding the status of invariance was obtained from the differences in fit indexes between stages. Accordingly, the significance of the Chi-Square differences between stages, the Comparative Fit Index (CFI), Tucker-Lewis Index (TLI), Root Mean Square Error of Approximation (RMSEA) and Standardized Root Mean Square Residual (SRMR) indices were examined according to the pre-established critical values. For the differences of ΔCFI and ΔTLI indices, the critical value was taken to be between ± 0.01 (F. F. Chen, 2007; Cheung & Rensvold, 2002; Hu & Bentler, 1999). Moreover, the critical values for ΔRMSEA and ΔSRMR were taken to be 0.015 and 0.030, respectively (Kline, 2018). The MGCFA analyses were conducted using R 4.2.1 (R Core

Team, 2022), with the dplyr (Wickham et al., 2023), corrplot (Taiyun & Viliam, 2021), lavaan (Rosseel, 2012) and semTools (Jorgensen et al., 2012) packages employed.

Ethics Statement

This study received approval from the Ethics Committee of Bolu Abant Izzet Baysal University (Approval Number: NO. 2021/430 [29.11.2021 and the meeting number: 2021/11]), and all procedures adhered to the ethical standards set by institutional and national research committees, along with the 1964 Helsinki Declaration and its subsequent amendments. Before participation, all participants received a written online information sheet outlining the study's goal, the voluntary nature of participation, data confidentiality, and the possibility to withdraw at any time without penalty. Participants electronically supplied written informed permission by actively signaling their acceptance using the online survey system prior to completing the questionnaire. Only the data from participants who granted this consent were incorporated into the study. This research did not include minors; hence, parental or guardian consent was unnecessary.

The recruitment period for this study was from 29 November 2021 to 25 December 2021.

RESULTS

This section presents the findings obtained in each step of the adjustment process. The results of the statistical analysis were interpreted in accordance with the theoretical findings. Although the presentations for some steps are not results, the information about those steps is provided as a heading to ensure completeness of all steps.

PC-1 (1) Obtain the necessary permission from the holder of the intellectual property rights relating to the test before carrying out any adaptation.

One of the authors involved in the development study was contacted by email to obtain permission to scale to this step. The email stated the purpose of this study and asked for permission. The author responded positively to the request.

PC-2 (2) Evaluate that the amount of overlap in the definition and content of the construct measured by the test and the item content in the populations of interest is sufficient for the intended use (or uses) of the scores.

The concept of transactional distance, particularly as represented by the new TDSTECH model, is of paramount importance for the comprehension of user engagement with

technology in distance education. As distance education programmes and their participants have expanded in recent years, the universality of these programmes has also increased. The similarity in the scale items indicates that the processes involved in distance education are consistent across different cultural contexts. This is evidenced by the similarity between the application methods and learners' feedback in both the source and target cultures. Distance education technologies exhibit a universal nature, transcending cultural differences. The significant overlap between the source and target cultures of the construct allows researchers to use participant scores interchangeably when studying similar cultural constructs.

PC-3 (3) Minimize the influence of any cultural and linguistic differences that are irrelevant to the intended uses of the test in the populations of interest.

In this step, the items of the scale were examined in detail for their content. A cultural and linguistic comparison was made. It was determined whether the content of the items needed to be updated according to the characteristics of the target culture. Thus, a linguistic and cultural equivalence comparison was made. Only "Moodle," one of the educational technology applications, was included in the items of the new TDSTECH. However, it was found that the distance education applications used in the target culture were different. Since applications such as LMS, Google Classroom, and MS Teams are used in addition to Moodle in the target culture, "Moodle" was changed to increase the awareness of the items among the participants. In Item 3, where participants first encountered Moodle, the phrase "online learning platforms (LMS, Moodle, MS Teams, Google Classroom, etc.)" was used instead. In other articles where Moodle appears, only the phrase "online learning platforms" was added by arranging it according to the sentence construct. After the application examples were given in the first use of the phrase, the part in parentheses was not added in the next articles in order not to decrease the motivation of the participants and not to increase the application time.

TD-1 (4) Ensure that the translation and adaptation processes consider linguistic, psychological, and cultural differences in the intended populations through the choice of experts with relevant expertise.

In this step, linguists familiar with the target languages were identified for the translation processes. A total of 13 linguists contributed to the translation processes. 2 linguists were consulted during the forward-backward translation process, 8 linguists were consulted during the post-forward translation evaluations, and 1 linguist was consulted during the final evaluation process.

TD-2 (5) Use appropriate translation designs and procedures to maximize the suitability of the test adaptation in the intended populations.

Appropriate translation designs were determined according to the method recommendation of Van De Vijver and Poortinga (2005) depending on the translatability of the scale. Accordingly, the similarities of the construct in cultural contexts and the items of the scale can be translated without significant changes. In this process, forward-backward translation processes were conducted and the opinions and recommendations of linguists were obtained between these processes. Based on these opinions and recommendations, the equivalence of the words was evaluated. In a final evaluation step, the words that were most appropriate for the target language were determined. The final step was to select the words whose meanings were most appropriate for the target culture, while maintaining the intended meaning. In this way, an attempt was made to ensure that the participants' perceived meanings of the terms in the source and target languages were similar. To verify similarity, an attempt was made to obtain statistical evidence through the analyses of the data from Study Group 1.

TD-3 (6) Provide evidence that the test instruetions and item content have similar meaning for all intended populations.

For this step, as mentioned in the previous step, a final evaluation was made with the assistance of an expert who is fluent in both languages and has lived for some time in the source and target cultures. During the evaluation process, the meanings of the words were discussed with the expert and the most appropriate words in the target language were selected. In addition to the final evaluation, as a continuation of the process in the previous step, a correlation analysis was performed between the scores obtained from the forwardand backward translation applications. The extent and direction of correlation between the scores of the two applications involving Study Group 1 were determined. The correlation coefficient between the results is +0.685. It can be interpreted that there is a positive and significant relationship close to the upper limit ($r_{xy} > 0.70$; $p < 0.05$) between the two application scores. These results and interpretations are statistical evidence that the content of the item and the test are similar for individuals in the source and target languages.

TD-4 (7) Provide evidence that the item formats, rating scales, scoring categories, test conventions, modes of administration, and other procedures are suitable for all intended populations.

Evidence that they are semantically identical in the target and source cultures indicates that the scale construct is appropriate for participants in the target culture. Furthermore, the scoring system of items and forms of application was conducted in the same way in the source culture.

TD-5 (8) Collect pilot data on the adapted test to enable item analysis, reliability assessment, and small-scale validity studies so that any necessary revisions to the adapted test can be made.

Item, reliability, and validity analyses of the scale were conducted on the data from Study Group 1. These analyses are preliminary. According to the results of these analyses, the item-total correlations in the scale ranged from 0.495 to 0.830, and it was interpreted that the item-total correlations were sufficient (> 0.300) (Field, 2009). In other words, the discrimination of the items in the form is at a sufficient level (> 0.300). In addition, the Cronbach Alpha reliability values calculated for the four-dimensional construct are 0.674, 0.938, 0.837, and 0.848, respectively. In the preliminary analyses for the scale, it can be concluded that the reliability values range from 0.60 to 0.70, which is an acceptable range (George & Mallery, 2003).

C-1 (9) Select sample with characteristics that are relevant for the intended use of the test and of sufficient size and relevance for the empirical analyses.

For validity and reliability analyses conducted in a larger and sufficient study group, the scale was applied to a group of 2402 students. After removing participants identified as outliers, the validity and reliability analyses were conducted on the data set of 2307 participants. Detailed definitions for this group can be found under the title Study Group 2 in the Method section.

C-2 (10) Provide relevant statistical evidence about the construct equivalence, method equivalence, and item equivalence for all intended populations.

In this step of the adaptation process, evidence was sought to ascertain the measurement invariance of the scale, employing three distinct categorical variables. Multi-group confirmatory factor analysis was utilised for this purpose. To this end, analyses were conducted on variables pertaining to gender, computer ownership, and unit variables. It should be noted that all variables used in these analyses are binary. Gender was divided into two subcategories: female and male. Computer ownership was dichotomised as present or absent. The unit variable was divided into four categories, representing two-year

undergraduate programmes. The results of the measurement invariance analyses for the three variables are presented in Table 4.

Table 4. Fit indexes of Multigroup Confirmatory Factor Analysis of Transactional Distance Scale

Variable	Fit Index	Configural Invariance	Metric Invariance	Scalar Invariance
Gender	Chi-Square	654.972*	664.597*	682.986*
	df	76	83	90
	RMSEA	0.082 (0.076 - 0.088)	0.079(0.073 - 0.085)	0.045 (0.039 - 0.050)
	CFI	0.969	0.968	0.968
	TLI	0.954	0.958	0.961
	SRMR	0.05	0.051	0.051
Possession of a personal computer	Chi-Square	667.395*	678.000*	705.256*
	df	76	83	90
	RMSEA	0.083 (0.077 - 0.089)	0.079(0.073 - 0.085)	0.078 (0.072 - 0.083)
	CFI	0.968	0.967	0.966
	TLI	0.953	0.957	0.959
	SRMR	0.05	0.051	0.052
Program	Chi-Square	661.176*	670.536*	691.705
	df	76	83	90
	RMSEA	0.082 (0.076 - 0.088)	0.079(0.073 - 0.085)	0.077 (0.071 - 0.082)
	CFI	0.968	0.968	0.967
	TLI	0.954	0.958	0.96
	SRMR	0.05	0.052	0.052

Table 4 presents the discrepancies in fit indices and significance values (p) for the chi-square obtained in a measurement invariance analysis for three variables. The analysis was conducted on three variables: gender, possession of a personal computer, and program. The data were examined across two levels of invariance comparisons. The configural, metric, and scalar invariance models were tested. The indices comprise Chi-Square (χ^2), p-values, Root Mean Square Error of Approximation (RMSEA), Comparative Fit Index (CFI), Tucker-Lewis Index (TLI), and Standardized Root Mean Square Residual (SRMR).

With regard to the variable of gender, the differences in the chi-square values are 9.625 ($p = 0.217$) for the configural-metric and 18.389 ($p = 0.009$) for the metric-scalar. The ΔRMSEA values demonstrate slight improvements, with differences of -0.003 and -0.034, respectively. The differences in the ΔCFI and ΔTLI are minimal, ranging from -0.001 to 0.004, indicating a stable fit across these levels. The ΔSRMR values remain largely unchanged, with differences of 0.001 and 0.000, respectively.

With regard to the possession of a personal computer, the differences in χ^2 values are 10.605 ($p = 0.168$) for Configural-Metric and 27.256 ($p = 0.003$) for Metric-Scalar. The ΔRMSEA

differences are -0.004 and -0.001, which indicate an improved fit at higher levels of invariance. The differences in the ΔCFI and ΔTLI values remain stable, ranging from -0.001 to 0.004. The ΔSRMR differences remain consistent at 0.001 for both comparisons.

With regard to the Program variable, the differences in χ^2 values are 9.360 ($p = 0.283$) for Configural-Metric and 21.169 ($p = 0.004$) for Metric-Scalar. The ΔRMSEA differences are -0.003 and -0.002, indicating an enhancement in the model's fit. The differences in the ΔCFI and ΔTLI are minimal, ranging from -0.001 to 0.004. The ΔSRMR differences are 0.002 and 0.000, indicating stable model fit across these levels.

These results indicate that while some significant changes in χ^2 values occur, particularly for the Metric-Scalar comparison, the overall fit indices (RMSEA, CFI, TLI, and SRMR) show minimal changes, thereby supporting the robustness of the measurement model across different levels of invariance. These findings align with the criteria suggested by F. F. Chen (2007) for assessing measurement invariance with small differences in fit indices.

C-3 (11) Provide evidence supporting the norms, reliability, and validity of the adapted version of the test in the intended populations.

To demonstrate the validity and reliability of the scale in the target language, a single-factor CFA and the calculated Cronbach's Alpha and HTMT2 values were shared with the data from study group 2. Item-total correlation values were also reported. The fit indices and interpretations that resulted from the CFA are shown in Table 4.

Table 5. Fit indexes of Transactional Distance Scale for target culture.

Fit Index	Range of Fit Indexes (Good&Intermediate fit)	Value	Interpretation
RMSEA	<0.08	0.079 (0.073 - 0.085)	Intermediate
CFI	>0.95	0.964	Good
TLI	>0.95	0.947	Good
SRMR	<0.06	0.049	Good
Chi-Square/df	<2	15.150	Poor
Chi-Square		575.673*	
df		38	

From the fit indices reported in Table 5, it can be inferred that the scale construct is similar in the source and target languages. These results are statistical evidence of the construct validity of the scale in the target culture. Accordingly, the transaction distance scale was found to be usable in Turkish culture. The path diagram obtained after CFA is shown in Figure 3.

Figure 3. Path diagram of the new TDSTECH in Turkish culture.

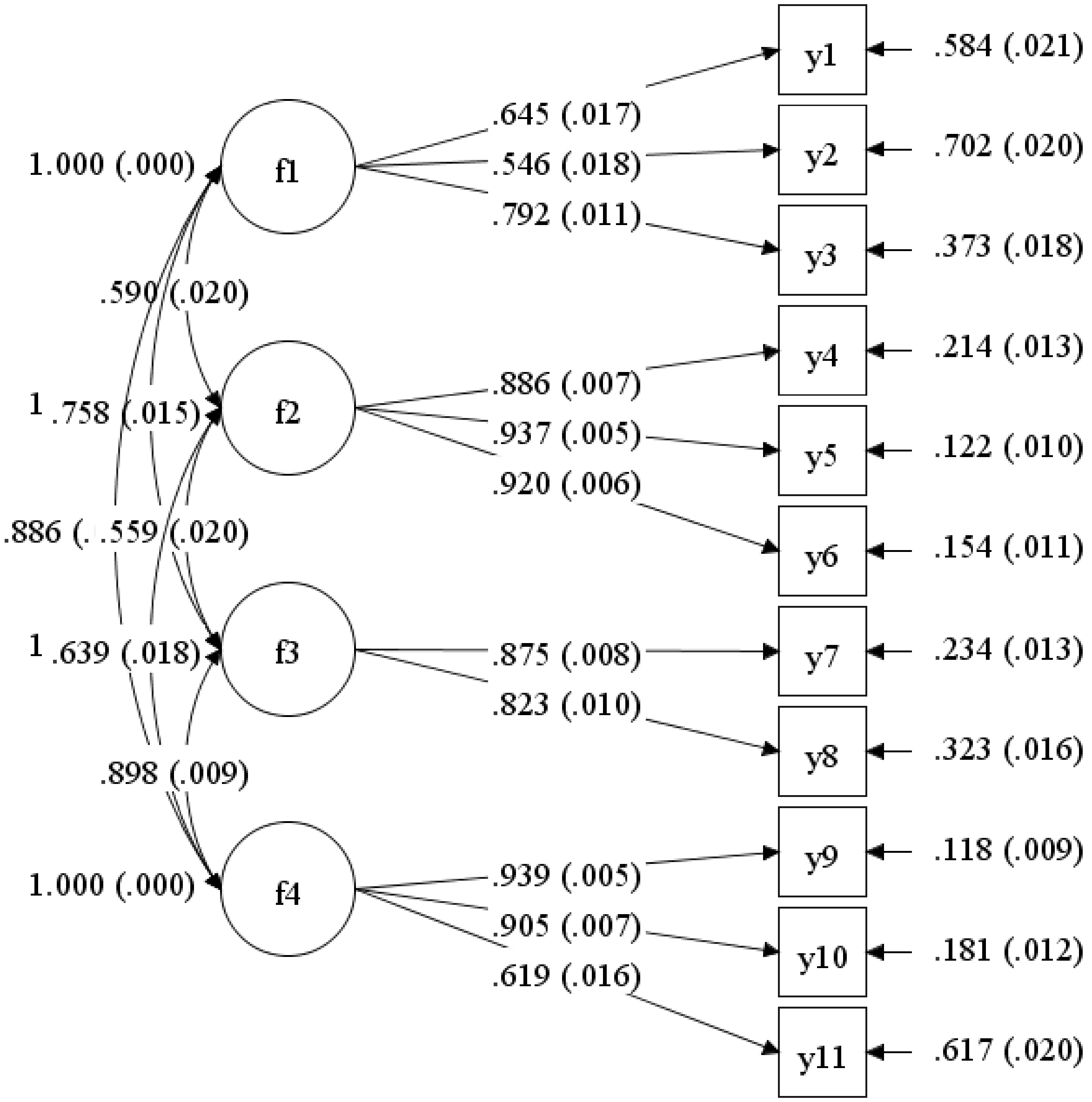

According to the results of CFA, it was found that the factor loadings/standard errors of the items ranged from 30.544 (y2) to 189.083 (y9). These results indicate that all items have a significant relationship at the 0.05 level (> 1.96) with the latent variables on which they depend. Another finding is that the factor loadings of the items vary between 0.546 (y2) and 0.939 (y9). The factor loadings were interpreted as appropriate (> 0.55; (Comrey & Lee, 1992)) when evaluated in light of the thresholds established for exploratory factor analysis. The variance ratios (R^2) explained by the items in the latent variables also ranged from 0.298-0.882. The highest factor loading and explained variance ratio were calculated for item 9, and

the lowest values were calculated for item 2. The Cronbach Alpha and HTMT2 coefficients calculated for the scale can be found in Table 5.

Table 6. Cronbach Alpha and HTMT2 values for Transactional Distance Scale for Study Group 2

Dimension	Cronbach Alpha		HTMT2			
			f1	f2	f3	f4
Learner Readiness For Using Technology (f1)	0.702	f1	1	0.601	0.746	0.879
Effectiveness (f2)	0.939	f2		1	0.556	0.743
Efficiency (f3)	0.837	f3			1	0.884
Satisfaction (f4)	0.852	f4				1

According to the results in Table 6, the reliability values of the scale dimensions ranged from 0.702 to 0.939. It was found that the learners' willingness to use technology dimension had the lowest reliability level and the effectiveness dimension had the highest reliability level. According to these results, it was interpreted that the reliability values of the dimensions are sufficient (> 0.70). When the HTMT2 for discriminant validity were evaluated, it was found that all dimensions of the scale met the threshold for discriminant validity (< 0.90). All HTML2 values were determined to be below 0.90.

In addition to these tests, the item-total correlations for Study Group 2 were also re-examined. It was found that the item-total correlations ranged from 0.495 to 0.830. The lowest correlation value was item 2; the highest correlation value was calculated for item 9. These results indicate that all items have a sufficient level to discriminate individuals (> 0.30).

C-4 (12) Use an appropriate equating design and data analysis procedures when linking score scales from different language versions of a test.

In this adaptation study, the application of the scale in different language versions was performed on the same group. Since the application was not performed on a group whose native language is different from the target language, this step is presented as a recommendation for future studies or projects.

A-1 (13) Prepare administration materials and instructions to minimize any culture- and language-related problems that are caused by administration procedures and response modes that can affect the validity of the inferences drawn from the scores.

To inform other researchers and potential participants about the process of using the adapted scale in new studies, a guidance text was created before the scale items. This text presents a brief definition of transaction distance, the marking procedure and scoring system of the items, the duration of the application, the silence procedure, and the contact information of the researchers who conducted the application.

A-2 (14) Specify testing conditions that should be followed closely in all populations of interest

It was found that there was no disability in using the scale as an online and/or as a paper-pencil form. However, considering that people with low computer literacy might have problems in marking in online forms, it is recommended to determine the application method according to the characteristics of the participant group. Another recommendation is not to provide all items in online forms for selection. Forcing a person to answer an item they do not want to answer may have a negative impact on their sincere views. It took 4-6 minutes to answer the questions, depending on the reading speed of the respondents. Along these lines, again taking into account the reading and marking speed of the participant group, one can allow 3-7 minutes for the application period.

SSI-1 (15) Interpret any group score differences with reference to all relevant available information.

In this step, the scores of students in study group 1 from both applications were compared using the T-test for independent samples. The aim is to provide further evidence for the linguistic equivalence of the scale. According to the results, it was found that the difference of 0.522 points between the arithmetic means calculated for both applications was not statistically significant: $t = 0.446$; $p > 0.05$. This result is further evidence that the forms in the source and target languages are equivalent in terms of language and meaning.

SSI-2 (16) Only compare scores across populations when the level of invariance has been established on the scale on which scores are reported.

As mentioned in Step 12, this study could not apply to a participant group other than those whose native language is the target language. Therefore, a recommendation for future studies and projects is made in this step.

Doc-1 (17) Provide technical documentation of any changes, including an account of the evidence obtained to support equivalence, when a test is adapted for use in another population.

A technical document was prepared in which tables were presented on the validity and reliability of the Quickfacts of the adaptation process. The technical report table was attached to the study as Appendix-1.

Doc-2 (18) Provide documentation for test users that will support good practice in the use of an adapted test with people in the context of the new population.

A brief application guide was prepared that included information on the application process and recommendations to consider. This guide is intended for researchers who wish to apply the scale in practice. The guide is provided as Appendix-2 at the end of the study.

CONCLUSION

The increasing number of distance learning programs has increased the importance of studies on the efficiency of the process. It is possible that the number of distance learning courses will increase much faster in the coming years, especially in view of the pandemic COVID -19 and the fact that distance learning has been preferred throughout the world for some time. Given this situation, determining the transaction distance of students benefiting from such programs with technology and intervening in situations where problems are identified will undoubtedly have a positive impact on the efficiency of the process. It is noted that one of the most important constructs to examine to ensure the effectiveness of distance education is transactional distance, and that low transactional distance contributes to meaningful learning (Anderson, 2003). Considering that these characteristics of distance education are universal, it is important to conduct these studies in Turkish culture.

The fact that the existing scales for determining transactional distance, which have been measured in many different studies using self-report, are outdated has led to the need to develop a new scale. In line with this need, the new TDSTECH was developed by Weidlich and Bastiaens (2018). The new TDSTECH, developed with the theory of transactional distance and the shortcomings of the existing scales in mind, is considered one of the most current measurement tools for this construct. Since transactional distance is an important construct in relation to distance education programs and the use of technology in the

instructional process, this study was conducted to adapt the new TDSTECH to Turkish culture.

In this study, considering the 18-step adaptation process recommended by the ITC, the linguistic equivalence of the scale was demonstrated through correlation and T-test analyzes. In addition, reliability analyzes conducted for the scale determined that the reliability values of the scale's four-dimensional structure were adequate. The CFA results were tested for construct validity. The results showed that the four-dimensional structure was compatible with the target culture. For discriminant validity, the evidence whose HTMT2 values were checked for each dimension was sufficient with this type of validity. All these results show that the new TDSTECH can be used in Turkish culture. It is believed that this adapted scale will contribute to the curriculum used in Turkey.

An important limitation of this study is that there was no cultural comparison of the scale. The fact that only students from the target culture were present when the scale was applied meant that situations such as test equality and item bias could not be investigated. For such studies, it is recommended that studies be conducted in collaboration with other researchers in different cultures.

References

Ali, M. M. (1987). Durbin–Watson and generalized Durbin–Watson tests for autocorrelations and randomness. *Journal of Business & Economic Statistics*, *5*(2), 195-203. https://doi.org/10.2307/139190

Altman, D. G., & Bland, J. M. (1995). Statistics notes: The normal distribution. *BMJ*, *310*(6975), 298. https://doi.org/10.1136/bmj.310.6975.298

Anderson, T. (2003). Getting the mix right again: An updated and theoretical rationale for interaction. *The International Review Of Research in Open and Distributed Learning*, *4*(2). https://doi.org/10.19173/irrodl.v4i2.149

Bollen, K. A. (1989). A new incremental fit index for general structural equation models. *Sociological Methods & Research*, *17*(3), 303-316. https://doi.org/10.1177/0049124189017003004

Breusch, T. S., & Pagan, A. R. (1979). A simple test for heteroscedasticity and random coefficient variation. *Econometrica*, 47(5), 1287-1294. https://doi.org/10.2307/1911963

Burbules, N. C. (1993). *Dialogue in teaching: Theory and practice.* New York, NY: Teachers College Press.

Chen, F. F. (2007). Sensitivity of goodness of fit indexes to lack of measurement invariance. *Structural Equation Modeling: A Multidisciplinary Journal*, *14*(3), 464-504. https://doi.org/10.1080/10705510701301834

Chen, Y. J. (2001). Dimensions of transactional distance in the world wide web learning environment: A factor analysis. *British Journal of Educational Technology*, *32*(4), 459-470. https://doi.org/10.1111/1467-8535.00213

Cheung, G. W., & Rensvold, R. B. (2002). Evaluating goodness-of-fit indexes for testing measurement invariance. *Structural Equation Modeling*, *9*(2), 233-255. https://doi.org/10.1207/s15328007sem0902_5

Cole, D. A. (1987). Utility of confirmatory factor analysis in test validation research. *Journal of Consulting and Clinical Psychology*, *55*(4), 584–594. https://doi.org/10.1037/0022-006X.55.4.584

Comrey, A. L., & Lee, H. B. (1992). Interpretation and application of factor analytic results. In A. L. Comrey & H. B. Lee (Eds) *A First Course in Factor Analysis* (2nd ed.) (pp. 240-262). Psychology Press. https://doi.org/10.4324/9781315827506

Croxton, R. A. (2014). The role of interactivity in student satisfaction and persistence in online learning. *Journal of Online Learning and Teaching, 10*(2), 314. URL: https://jolt.merlot.org/vol10no2/croxton_0614.pdf

Cudeck, R., & Browne, M. W. (1983). Cross-validation of covariance structures. *Multivariate Behavioral Research*, *18*(2), 147-167. https://doi.org/10.1207/s15327906mbr1802_2

De Maesschalck, R., Jouan-Rimbaud, D., & Massart, D. L. (2000). The Mahalanobis distance. *Chemometrics and Intelligent Laboratory Systems*, *50*(1), 1-18. https://doi.org/10.1016/s0169-7439(99)00047-7

Durbin, J., & Watson, G. S. (1950). Testing for serial correlation in least squares regression: I. *Biometrika*, 37(3-4), 409-428. https://doi.org/10.2307/2332391

Ekwunife-Orakwue, K. C., & Teng, T. L. (2014). The impact of transactional distance dialogic interactions on student learning outcomes in online and blended environments. *Computers & Education*, *78*, 414-427. https://doi.org/10.1016/j.compedu.2014.06.011

Ergonomic requirements for office work with visual display terminals (VDTs) (n.d.). https://doi.org/10.3403/01822507u

Field, A. (2009). *Discovering Statistics Using IBM SPSS Statistics (Third edition)*. Sage Publications.

Gana, K., & Broc, G. (2018). Structural Equation Modeling with lavaan. https://doi.org/10.1002/9781119579038

George, D., & Mallery, P. (2003). *SPSS for Windows step by step: A simple guide and reference* (4th ed.). Allyn & Bacon.

Ghasemi, A., & Zahediasl, S. (2012). Normality tests for statistical analysis: a guide for non-statisticians. *International Journal of Endocrinology and Metabolism*, *10*(2), 486-489. https://doi.org/10.5812/ijem.3505

Goel, L., Zhang, P., & Templeton, M. (2012). Transactional distance revisited: Bridging face and empirical validity. *Computers in Human Behavior*, *28*(4), 1122-1129. https://doi.org/10.1016/j.chb.2012.01.020

Gold, A. H., Malhotra, A., & Segars, A. H. (2001). Knowledge management: an organizational capabilities perspective. *Journal of Management Information Systems, 18*(1), 185–214. https://doi.org/10.1080/07421222.2001.11045669

Halunga, A. G., Orme, C. D., & Yamagata, T. (2017). A heteroskedasticity robust Breusch–Pagan test for Contemporaneous correlation in dynamic panel data models. *Journal of Econometrics*, *198*(2), 209-230. https://doi.org/10.1016/j.jeconom.2016.12.005

Hambleton, R. K., & Patsula, L. (1999). Increasing the validity of adapted tests: Myths to be avoided and guidelines for improving test adaptation practices. *Journal of Applied Testing Technology*, *1*(1), 1-30. URL: https://www.semanticscholar.org/paper/Increasing-the-Validity-of-Adapted-Tests%3A-Myths-to-Hambleton-Patsula/0ae7b654489e792633a51f655607db772fbce8f8

Hillman, D. C. A., Willis, D. J., & Gunawardena, C. N. (1994). Learner-interface interaction in distance education: An extension of contemporary models and strategies for practitioners. *American Journal of Distance Education*, *8*(2), 30-42. https://doi.org/10.1080/08923649409526853

Horzum, M. B. (2011). Developing transactional distance scale and examining transactional distance perception of blended learning students in terms of different variables. *Educational Sciences: Theory and Practice*, *11*(3), 1582-1587. URL: https://eric.ed.gov/?id=EJ936610

Hu, L.-t., & Bentler, P. M. (1999). Cutoff criteria for fit indexes in covariance structure analysis: Conventional criteria versus new alternatives. *Structural Equation Modeling: A Multidisciplinary Journal*, *6*(1), 1-55. https://doi.org/10.1080/10705519909540118

Hung, M.-L., Chou, C., Chen, C.-H., & Own, Z.-Y. (2010). Learner readiness for online learning: Scale development and student perceptions. *Computers & Education*, *55*(3), 1080-1090. https://doi.org/10.1016/j.compedu.2010.05.004

ITC Guidelines for Translating and Adapting Tests (Second Edition) (2018). *International Journal of Testing*, *18*(2), 101-134. https://doi.org/10.1080/15305058.2017.1398166

Joo, Y. J., Lim, K. Y., & Kim, E. K. (2011). Online university students' satisfaction and persistence: Examining perceived level of presence, usefulness and ease of use as predictors in a structural model. *Computers & Education, 57*(2), 1654-1664. https://doi.org/10.1016/j.compedu.2011.02.008

Jorgensen, T. D., Pornprasertmanit, S., Schoemann, A. M., & Rosseel, Y. (2012). semTools: Useful Tools for Structural Equation Modeling. CRAN: Contributed Packages. https://doi.org/10.32614/cran.package.semtools

Kauffman, H. (2015). A review of predictive factors of student success in and satisfaction with online learning. *Research in Learning Technology, 23*, 26507. https://doi.org/10.3402/rlt.v23.26507

Kline, R. B. (2018). Response to Leslie Hayduk's Review of Principles and Practice of Structural Equation Modeling, 4th Edition. *Canadian Studies in Population, 45*(3-4), 188. https://doi.org/10.25336/csp29418.

Koh, J. H. L., & Daniel, B. K. (2022). Shifting online during COVID-19: A systematic review of teaching and learning strategies and their outcomes. *International Journal of Educational Technology in Higher Education, 19*(1), 56. https://doi.org/10.1186/s41239-022-00361-7

Lee, Y., & Choi, J. (2013). A structural equation model of predictors of online learning retention. *The Internet and Higher Education, 16*, 36-42. https://doi.org/10.1016/j.iheduc.2012.01.005

Li, C.-H. (2016). The performance of ML, DWLS, and ULS estimation with robust corrections in structural equation models with ordinal variables. *Psychological Methods, 21*(3), 369-387. https://doi.org/10.1037/met0000093

Mardia, K. V. (1974). Applications of some measures of multivariate skewness and kurtosis in testing normality and robustness studies. Sankhyā: *The Indian Journal of Statistics, Series B*, 115-128. URL: https://www.jstor.org/stable/25051892

Meredith, W. (1993). Measurement invariance, factor analysis and factorial invariance. *Psychometrika, 58*(4), 525-543. https://doi.org/10.1007/BF02294825

Moore, M. G. (2018). The Theory of Transactional Distance. *Handbook of Distance Education*, 32-46. https://doi.org/10.4324/9781315296135-4

Moore, S., & Hodges, C. (2023). Emergency Remote Teaching. *EdTechnica*. https://doi.org/10.59668/371.11681

Osborne, J. W., & Waters, E. (2002). Four assumptions of multiple regression that researchers should always test. *Practical Assessment, Research & Evaluation*, *8*(2), 1-9. URL: https://openpublishing.library.umass.edu/pare/article/1461/galley/1412/view/

Paul, R. C., Swart, W., Zhang, A. M., & MacLeod, K. R. (2015). Revisiting Zhang's scale of transactional distance: Refinement and validation using structural equation modeling. *Distance Education*, *36*(3), 364-382. https://doi.org/10.1080/01587919.2015.1081741

Peters, O. (2013). Learning and Teaching in Distance Education. https://doi.org/10.4324/9780203046029

Rademaker, M. E., & Schuberth, F. (2020). cSEM: Composite-Based Structural Equation Modeling. CRAN: Contributed Packages. https://doi.org/10.32614/cran.package.csem

R Core Team (2022). R: A language and environment for statistical computing. R Foundation for Statistical Computing, Vienna, Austria. URL: https://www.R-project.org/.

Roemer, E., Schuberth, F., & Henseler, J. (2021). HTMT2–an improved criterion for assessing discriminant validity in structural equation modeling. *Industrial Management & Data Systems*, *121*(12), 2637-2650. https://doi.org/10.1108/imds-02-2021-0082

Rosseel, Y. (2012). lavaan: An R Package for Structural Equation Modeling. *Journal of Statistical Software, 48*(2), 1-36. https://doi.org/10.18637/jss.v048.i02

Sato, S. N., Condes Moreno, E., Rubio-Zarapuz, A., Dalamitros, A. A., Yañez-Sepulveda, R., Tornero-Aguilera, J. F. & Clemente-Suárez, V. J. (2024). Navigating the new normal: Adapting online and distance learning in the post-pandemic era. *Education Sciences, 14*(1), 19. https://doi.org/10.3390/educsci14010019

Satorra, A., & Bentler, P. M. (1994). Corrections to test statistics and standard errors in covariance structure analysis. In A. von Eye & C. C. Clogg (Eds.), *Latent variables analysis: Applications for developmental research* (pp. 399-419). Sage Publications. URL: https://psycnet.apa.org/record/1996-97111-016

Sumner, J. (2000). Serving the system: A critical history of distance education. *Open Learning: The Journal of Open, Distance and e-Learning*, *15*(3), 267-285. https://doi.org/10.1080/713688409

Tabachnick, B. G., Fidell, L. S., & Ullman, J. B. (2013). *Using multivariate statistics* (Vol. 6, pp. 497-516). Boston, MA: Pearson.

Taiyun, W., & Viliam, S. (2021). R package 'corrplot': Visualization of a Correlation Matrix (Version 0.92). URL: https://github.com/taiyun/corrplot

Teo, T. S. H., Srivastava, S. C., & Jiang, L. (2008). Trust and electronic government success: an empirical study. *Journal of Management Information Systems, 25*(3), 99–132. https://doi.org/10.2753/mis0742-1222250303

Van De Vijver, F. J. R., & Poortinga, Y. H. (2005). Conceptual and Methodological Issues in Adapting Tests. in *Adapting educational and psychological tests for cross-cultural assessment* .Hambleton, R. K., Merenda, P. F., & Spielberger, C. D. (Eds.). New Jersey: Lawrence Erlbaum Associates, Publishers.

Weidlich, J., & Bastiaens, T. J. (2018). Technology matters - The Impact of Transactional Distance on Satisfaction in Online Distance Learning. *The International Review of Research in Open and Distributed Learning, 19*(3). https://doi.org/10.19173/irrodl.v19i3.3417

Wickham, H., Francois, R., Henry, L., Muller, K., & Vaughan, D. (2023). _dplyr: A Grammar of Data Manipulation_. R package version 1.1.4, URL:https://CRAN.R-project.org/package=dplyr

Yılmaz, R., & Keser, H. (2015). İşlemsel Uzaklık Ölçeğinin Uyarlama Çalışması [The Adaptation Study of Transactional Distance Scale]. *Hacettepe Üniversitesi Eğitim Fakültesi Dergisi [Hacettepe University Journal of Education]*, *30*(4), 91-105. URL: http://www.efdergi.hacettepe.edu.tr/shw_artcl-1775.html

Zhang, A. M. (2003). *Transactional distance in web-based college learning environments: Toward measurement and theory construction* (Unpublished doctoral dissertation). Commonwealth University, Virginia. URL: https://www.proquest.com/openview/56aa7eadfe2aaafa4d088c082f37a429/1?pq-origsite=gscholar&cbl=18750&diss=y

Appendix

Click here to access/download
Supporting Information
Appendix.docx

R code

Click here to access/download

Supporting Information - Compressed/ZIP File Archive

R code.rar

Click here to access/download
Supporting Information - Compressed/ZIP File Archive
Mplus File.rar
