# Supplementary material for: Turkish adaptation of a new scale for measuring transactional distance between students and the learning technology
Source: PLoS One. 2025 Sep 24;20(9):e0331789. doi: 10.1371/journal.pone.0331789 (PMC12459776; doi:10.1371/journal.pone.0331789)
Supplement: Attachment — Submitted filename: Response to Reviewers.docx [file pone.0331789.s005.docx]

**Response to Reviewers**

Dear Editor,

We are pleased to submit the revised version of our manuscript entitled “Turkish Adaptation of a New Scale for Measuring Transactional Distance Between Students and the Learning Technology” for further consideration by PLOS ONE.

We would like to express our sincere gratitude to you and the reviewers for your constructive comments and detailed feedback. We have carefully reviewed each point raised and implemented all recommended changes to improve the clarity, readability, and overall quality of the manuscript. The revision process involved:

- Thorough professional English language editing by using Quilbot Premium.
- Full alignment with PLOS ONE formatting and style guidelines.
- Structural revisions to the Abstract, Introduction, Methods, Results, Discussion, and Conclusion.
- Comprehensive responses to reviewer and editor comments, as documented in the attached point-by-point table.
- Upload of all datasets, R and Mplus code files, and supplementary documents to Zenodo.
- All relevant data are available in the Zenodo repository: Yandı, A. (2025). Turkish Adaptation of a New Scale for Measuring Transactional Distance Between Students and the Learning Technology [Data set]. Zenodo. <https://doi.org/10.5281/zenodo.16880975>
- We believe that these revisions address all concerns raised and have significantly strengthened the manuscript, enhancing both methodological rigor and clarity of presentation.

We appreciate the opportunity to revise our work and look forward to your favorable consideration.

Sincerely,

Dr. Alperen Yandı

Bolu Abant Izzet Baysal University

Faculty of Education, Department of Educational Measurement and Evaluation

# Point-by-Point Response Table

| Reviewer / Editor Comment | Author Response / Action Taken |
| --- | --- |
| PLOS ONE style compliance: Ensure manuscript follows journal style, including file naming, reference formatting, and section order. | Revised the manuscript fully in accordance with PLOS ONE style templates. Corrected section headings, table and figure captions, reference style (numbers in square brackets), and removed any sections not in journal structure. Updated file names accordingly. |
| Data Availability Statement incomplete: Repository name and DOI missing. | All datasets, R and Mplus syntax files, and supplementary documents have been deposited in Zenodo. Updated Data Availability Statement to read: “All relevant data are available in the Zenodo repository: Yandı, A. (2025). Turkish Adaptation of a New Scale for Measuring Transactional Distance Between Students and the Learning Technology [Data set]. Zenodo. https://doi.org/10.5281/zenodo.16880975.” |
| Data sharing plan clarification: Entire dataset must be accessible upon acceptance. | Confirmed full open access to anonymized datasets and analysis scripts in Zenodo. Statement revised to reflect compliance with PLOS ONE open data policy. |
| Ethics statement placement: Must only appear in Methods section. | Ethics statement relocated to the Methods section exclusively. Removed from all other parts of the manuscript. |
| Language and clarity: Comprehensive editing by a native/professional editor required. | The entire manuscript underwent professional English language editing to improve grammar, sentence structure, clarity, and remove redundancies. |
| Abstract – avoid repetition, include key statistical results, remove citation of original scale. | Abstract rewritten to remove repetitive content, add key statistical indicators (e.g., CFI, TLI, RMSEA, SRMR, Cronbach’s α), and exclude any mention of the original scale. |
| Title – avoid acronyms such as TDSTECH. | Title revised to spell out the full name: “Turkish Adaptation of a New Scale for Measuring Transactional Distance Between Students and the Learning Technology.” |
| Remove Biography section (not part of PLOS ONE structure). | Biography section completely removed. |
| Introduction too long – improve paragraph structure and conciseness. | Introduction shortened by removing redundant sentences, reorganized into shorter, coherent paragraphs with smoother transitions. |
| Aim statement should be short and clear. | Rephrased the aim into a single concise sentence at the end of the Introduction. |
| Method section – move data collection period to beginning. | Added the data collection period (Nov 29–Dec 25, 2021) at the start of the Method section. |
| Method section – streamline overly detailed descriptions. | Condensed lengthy procedural descriptions while keeping essential methodological information; reduced tedious procedural repetition. |
| Clearly explain sample size determination. | Added literature-based justification for sample size, citing MacCallum et al. (1999), Wolf et al. (2013), and Kline (2018). |
| Move CFA results from Method to Results section. | Relocated all CFA model fit results, factor loadings, and related analysis details to the Results section. |
| Clarify “Scientiæ Baccalaureus” term. | Updated term to “Bachelor’s degree students.” |
| Blank page (p. 29) in manuscript. | Deleted the blank page. |
| CFA χ²/df ratio high – discuss. | Discussion now explains sensitivity of χ²/df to large sample sizes and emphasizes evaluation using alternative fit indices (CFI, TLI, RMSEA, SRMR). |
| No Discussion section – include comparisons, implications, and limitations. | Added a full Discussion section comparing findings to prior literature, interpreting model fit, and outlining both practical implications and study limitations. |
| Conclusion too brief – extend beyond summary. | Conclusion expanded to highlight significance of findings, implications for practice, and recommendations for future research. |
| Limitations and future research suggestions should be developed. | Expanded limitations to include lack of multilingual samples, university-only sample, and contextual constraints; added explicit recommendations for future work. |
| Clarify “student-interface interaction” in Turkish distance education context. | Added explanation in Introduction and Discussion contextualizing this concept in Turkey’s distance education culture with relevant examples. |
| Ensure references follow PLOS ONE style and citation numbering. | Revised all in-text citations to numerical format; reformatted reference list per PLOS ONE guidelines, verifying and adding DOIs where available. |
| Check relevance of suggested citations. | Reviewed all suggested works; included only those directly relevant to the study’s aims and context. |
| Technical checks (figures/tables). | Reformatted all tables and figures to comply with journal layout, numbering, and caption standards. |
| Reviewer #1 – Replace “Moodle” with contextually appropriate platforms. | Replaced “Moodle” with “online learning platforms (e.g., LMS, Moodle, MS Teams, Google Classroom)” in relevant items. |
| Reviewer #2 – Explain ‘student-interface interaction’ term in detail. | Provided clear definition and examples tailored to Turkish online education context, illustrating its significance for learner engagement. |
